# *Sinularia leptoclados* (Ehrenberg, 1834) (Cnidaria, Octocorallia) re-examined

**DOI:** 10.3897/zookeys.272.4406

**Published:** 2013-02-25

**Authors:** Leen P. van Ofwegen, Yehuda Benayahu, Catherine S. McFadden

**Affiliations:** 1 Department of Marine Zoology, Naturalis Biodiversity Center, P.O. Box 9517, 2300 RA Leiden, the Netherlands; 2 Department of Zoology, George S. Wise Faculty of Life Sciences, Tel Aviv University, Ramat Aviv, 69978, Israel; 3 Department of Biology, Harvey Mudd College, Claremont, CA 91711, USA

**Keywords:** Alcyonacea, re-description, new species, Indo-Pacific, Red Sea, taxonomy, phylogeny

## Abstract

*Sinularia leptoclados* (Ehrenberg, 1834) is re-described. *Sinularia leptoclados* var. *gonatodes* Kolonko, 1926 is synonymized with *Sinularia maxima* Verseveldt, 1977. Two new species of *Sinularia* with digitiform lobules, *leptoclados*-type surface clubs and unbranched interior spindles, are described. An updated maximum likelihood tree of *Sinularia* species with *leptoclados*-type clubs (clade 5C) based on two mitochondrial genes (*mtMutS*, *COI*) and a nuclear gene (28S rDNA) is presented.

## Introduction

In his revision of the soft coral genus *Sinularia*, Verseveldt (1980) mentioned three stalked *Sinularia* species with digitiform lobules, *leptoclados*-type surface clubs and unbranched interior spindles. These are *Sinularia firma* Tixier-Durivault, 1970, *Sinularia leptoclados* (Ehrenberg, 1834), and *Sinularia maxima* Verseveldt, 1971. Subsequently, comparison of material collected from New Caledonia (RMNH Coel. 10447-10449) to type material proved *Sinularia firma* to be an encrusting species. The original description of *Sinularia firma* was based on a tiny fragment that obscured the colony growth form. In the key of Verseveldt (1980: 12) *Sinularia leptoclados* and *Sinularia maxima* were separated from each other by colony growth form, *Sinularia maxima* with robust lobes, up to 120 mm high, and *Sinularia leptoclados* with shorter ones. Verseveldt based his description of *Sinularia leptoclados* on a RMNH specimen from the Red Sea, without mentioning its catalogue number. He certainly did not have the type specimen, as he states that he failed to find that in museum collections (Verseveldt: 9). Additionally, in his revision Verseveldt synonymized *Sinularia leptoclados* var. *gonatodes* Kolonko, 1926, with *Sinularia leptoclados* and stated the species exhibited an Indo-Pacific distribution.

The first two authors have based their identifications of *Sinularia leptoclados* on the microscope slides of Verseveldt at their disposal, and following the *Sinularia* revision of Verseveldt (1980), have considered *Sinularia leptoclados* specimens to be stalked with finger-like lobules and variable *leptoclados*-type clubs in the surface layer of the colony. The results have been published in a series of studies (see below) that have further supported Verseveldt’s (1980) statement that the species is widespread in the Red Sea and in the Indo-West Pacific area.

Alderslade and Shirwaiker (1991) were the first after Verseveldt’s (1980) revision to describe another species with characterssimilar to *Sinularia leptoclados*, their *Sinularia kavarattiensis* from the Laccadive Archipelago, India. They compared *Sinularia kavarattiensis* with the holotype of *Sinularia leptoclados* var. *gonatodes* and considered the many small spindles present in the surface layer of the lobes of the latter asa major difference between the two species. Later on, Manuputty and Ofwegen (2007) described three species from Ambon (Indonesia) which resembled *Sinularia leptoclados*: *Sinularia acuta*, *Sinularia corpulentissima* and *Sinularia longula*. In that studythey used for comparison a specimen from Ambon (RMNH Coel. 38426), considered by them to be *Sinularia leptoclados*.

McFadden et al. (2009), the first molecular study of the genus *Sinularia* ever conducted, discovered that specimens from Australia identified as *Sinularia leptoclados* by P. Alderslade (NTM C5421) and the first author (NTM C14492, 14519-21) differed genetically from Red Sea specimens identified as *Sinularia leptoclados* by the second author (ZMTAU CO 34095). This unexpected finding prompted us to re-examine the *Sinularia leptoclados* collections of the RMNH and ZMTAU and to search for the type material of this widespread species (e.g., Verseveldt). Fortunately, we discovered the type specimen of *Sinularia leptoclados* still exists in the ZMB, probably overlooked by Verseveldt, while revising the genus, most likely because it was labelled as *Lobularia leptoclados* Ehrenberg, 1834. After examination of its sclerites and comparison to RMNH and ZMTAU material identified as *Sinularia leptoclados* it became obvious that this species does not exhibit an Indo-West Pacific distribution as stated by Verseveldt (1980), but is rather limited to the Red Sea and eastern Indian Ocean. Material wrongly assigned to *Sinularia leptoclados* from other parts of the Indo-Pacific by the two first authors proved to be a mixture of misidentifications and as yet undescribed species. Interestingly, the specimens from Australia that were erroneously identified as *Sinularia leptoclados* have sclerites and a colony morphology that closely resemble that species. However, certain small morphological differences, its unique genetic haplotype, and the now disjunct distribution (Red Sea and western Indian Ocean *vs*. Pacific Ocean, Australia), convinced us to describe this material as a new species.

While collecting new material of *Sinularia leptoclados* at Eilat, northern Gulf of Aqaba, Red Sea, we unexpectedly found two other species with *leptoclados*-type clubs and *leptoclados*-like colony shape: *Sinularia verseveldti* Ofwegen, 1996 (Fig. 5f), so far only known from the Pacific, and a yet undescribed species which is described here.

## Material and methods

### Morphological examination

In order to identify the material, sclerites from different parts of the colony were obtained by dissolving the tissues in 10% sodium hypochlorite, followed by rinsing in fresh water. When appropriate, they were prepared for scanning electron microscopy as follows: the sclerites were carefully rinsed with double-distilled water, dried at room temperature, coated with gold and examined with a Jeol 6480LV electron microscope, operated at 10 kV.

Material studied is deposited in the Naturalis Biodiversity Center (formerly Rijks-museum van Natuurlijke Historie, Leiden, the Netherlands (RMNH)), Zoological Museum, Department of Zoology, Tel Aviv University, Israel (ZMTAU), Museum für Naturkunde der Humboldt-Universität, Berlin, Germany (ZMB), Zoological Reference Collection (ZRC) of the Raffles Museum of Biodiversity Research, Singapore, and the Museum and Art Gallery of the Northern Territory, Darwin, Australia (NTM).

### Molecular phylogenetic analysis

Extraction of DNA from ethanol-preserved tissue samples, PCR amplification, and sequencing of the *mtMutS* (*msh1*), *COI* and 28S rDNA genes followed the protocols published in McFadden et al. (2011) and McFadden and Ofwegen (2012). Sequence data were proofread using LaserGene software, and aligned using the L-INS-i method in MAFFT (Katoh et al. 2005). Pairwise measures of genetic distance (uncorrected p) among sequences were computed using MEGA v.5 (Tamura et al. 2011). Modeltest 3.0 (Posada and Crandall 1998) was used to select appropriate models of evolution for maximum likelihood analyses that were run using GARLI 2.0 (Zwickl 2006). Trees for *mtMutS* and *COI* were generally congruent with those for 28S rDNA, so in addition to separate analyses of the mitochondrial and nuclear genes we also ran a combined analysis with different models of evolution applied to each data partition (*mtMutS* + *COI*: TrN+I; *28S*: GTR+I+G). Bayesian analyses of the same separate and combined data sets were run using MrBayes v. 3.2.1 (Ronquist et al. 2012) and a GTR+I+G model of evolution applied to both partitions; analyses were run for 2 million generations (until standard deviation of split partitions < 0.01) with a burn-in of 25% and default Metropolis coupling parameters. We included in our analyses all other species from *Sinularia* clade 5C for which sequence data were available for at least two of the three genes (Table 1); three species belonging to clades 5A (club sclerites with a distinct central wart, polyps with collaret, points and tentacle scales; *Sinularia gardineri*) and 5B (club sclerites with a distinct central wart, polyps with collaret, points and tentacle rods; *Sinularia hirta*, *Sinularia terspilli*) were used as outgroup taxa.

**Table 1.** Specimens of *Sinularia* included in the molecular phylogenetic analyses. NTM = Museum and Art Gallery of the Northern Territory; RMNH = Naturalis Biodiversity Center; ZMTAU = Zoological Museum, Tel Aviv University. Bold = new GenBank accessions; NA = no sequence obtained.

		GenBank Acc. No.		
Species	Museum Acc. No.	*COI*	*mtMutS*	*28S rDNA*
*Sinularia abrupta*	NTM C14012	**KC542862**	**KC542849**	NA
*Sinularia abrupta*	ZMTAU Co 33623	JX991256	JX991168	**KC542822**
*Sinularia acuta*	RMNH Coel. 38721	**KC542863**	**FJ621376**	NA
*Sinularia acuta*	ZMTAU Co 33617	JX991257	JX991169	**KC542823**
*Sinularia australiensis* sp. n.	NTM C14492	**KC542864**	FJ621437	**KC542824**
*Sinularia australiensis* sp. n.	NTM C14519	**KC542865**	FJ621438	**KC542825**
*Sinularia bisulca*	RMNH Coel. 38724	**KC542866**	FJ621378	**KC542826**
*Sinularia corpulentissima*	RMNH Coel. 40839	**KC542867**	KC542850	**KC542827**
*Sinularia daii*	ZMTAU Co 34665	JX991258	JX991170	**KC542828**
*Sinularia densa*	RMNH Coel. 40840	**KC542868**	**KC542851**	**KC542829**
*Sinularia digitata*	RMNH Coel. 40841	**KC542869**	**KC542852**	**KC542830**
*Sinularia eilatensis* sp. n.	ZMTAU Co 35260	**KC542870**	**KC542853**	**KC542831**
*Sinularia eilatensis* sp. n.	ZMTAU Co 35305	**KC542873**	**KC542856**	**KC542834**
*Sinularia ?eilatensis* sp. n.	ZMTAU Co 35303	**KC542871**	**KC542854**	**KC542832**
*Sinularia ?eilatensis* sp. n.	ZMTAU Co 35304	**KC542872**	**KC542855**	**KC542833**
*Sinularia erecta*	ZMTAU Co 34144	GU355981	FJ621404	**KC542835**
*Sinularia gardineri* (5A)	ZMTAU Co 34097	GU355982	FJ621414	**KC542819**
*Sinularia hirta* (5B)	ZMTAU Co 34100	GU355983	FJ621428	**KC542820**
*Sinularia leptoclados*	ZMTAU Co 35308	**KC542874**	**KC542857**	**KC542836**
*Sinularia leptoclados*	ZMTAU Co 34095	GU355980	FJ621439	**KC542837**
*Sinularia longula*	RMNH Coel. 38439	**KC542875**	FJ621441	**KC542838**
*Sinularia maxima*	NTM C14512	**KC542876**	FJ621448	**KC542839**
*Sinularia molesta*	RMNH Coel. 38440	**KC542877**	FJ621449	NA
*Sinularia penghuensis*	ZMTAU Co 34659	JX991273	JX991183	**KC542840**
*Sinularia penghuensis*	ZMTAU Co 34681	JX991274	JX991184	**KC542841**
*Sinularia penghuensis*	ZMTAU Co 34739	JX991276	JX991186	**KC542842**
*Sinularia robusta*	NTM C14518	**KC542878**	FJ621473	**KC542843**
*Sinularia slieringsi*	ZMTAU Co 34654	JX991277	JX991187	NA
*Sinularia terspilli* (5B)	ZMTAU Co 34156	GU355984	FJ621481	**KC542821**
*Sinularia verseveldti*	ZMTAU Co 35309	**KC542879**	**KC542858**	**KC542844**
*Sinularia verseveldti*	RMNH Coel. 40842	**KC542880**	**KC542859**	**KC542845**
*Sinularia verseveldti*	RMNH Coel. 40843	**KC542881**	**KC542860**	**KC542846**
*Sinularia verseveldti*	RMNH Coel.40844	**KC542882**	**KC542861**	**KC542847**
*Sinularia wanannensis*	ZMTAU Co 34704	JX991281	JX991190	**KC542848**

## Taxonomy

### Sinularia australiensis sp. n.

urn:lsid:zoobank.org:act:C0EC77D7-A9DF-49A6-8BC4-C93AC3AFE8AF

http://species-id.net/wiki/Sinularia_australiensis

**Figure 1.**
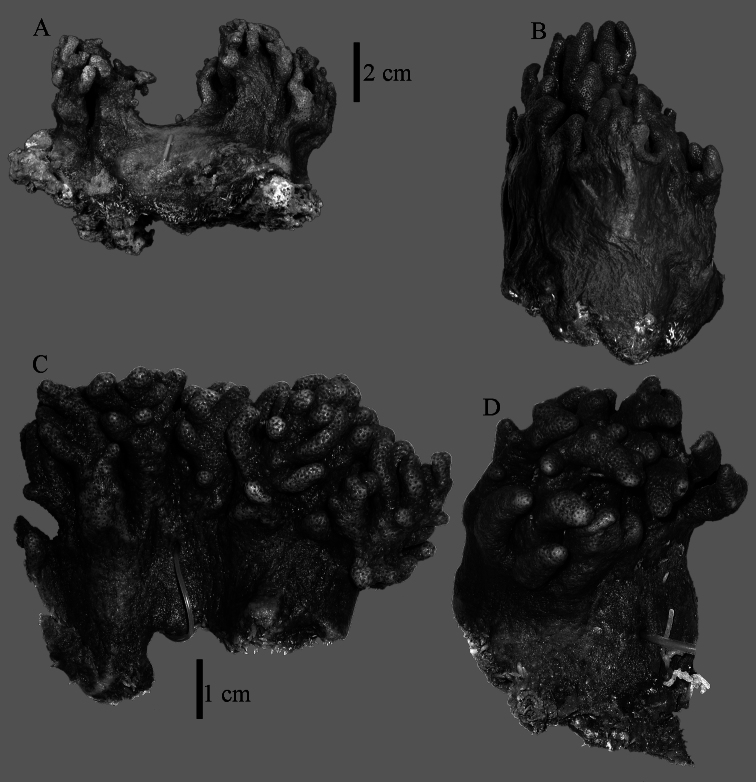
*Sinularia australiensis* sp. n., **A** holotype NTM C14519 **B** paratype NTM C14492 **C** paratype NTM C14520 **D** paratype NTM C14521. Scale at **A** also applies to **B**, scale at **C** also to **D**.

Figs 1 [Fig F2] [Fig F3] –4

? Sinularia leptoclados ; Lüttschwager, 1915: 3 (West Australia); Macfadyen: 37 (Great Barrier Reef Australia).
Sinularia leptoclados ; Ofwegen, 2008a: 131; McFadden et al.: 320 (Gulf of Carpentaria, West Australia).

#### Material examined.

Holotype: NTM C14519, Australia, Northern Territory, Gulf of Carpentaria, West of Bremer island, 12°05.660'S, 136°47.754'E, depth 1–3 m, coll. P. Alderslade & party, 17 December 2003. Paratypes: NTM C14492,C14520, C14521, same data as holotype.

#### Description.

The holotype is 6 cm high and 9.5 cm wide, attached to a piece of rock (Fig. 1A). The middle part of the colony is devoid of lobes, possibly a colony in the process of colony fission. The primary lobes branch off once or twice, lobules knob- to finger-shaped, up to 4 mm wide and 1 cm long.

**Figure 2. F2:**
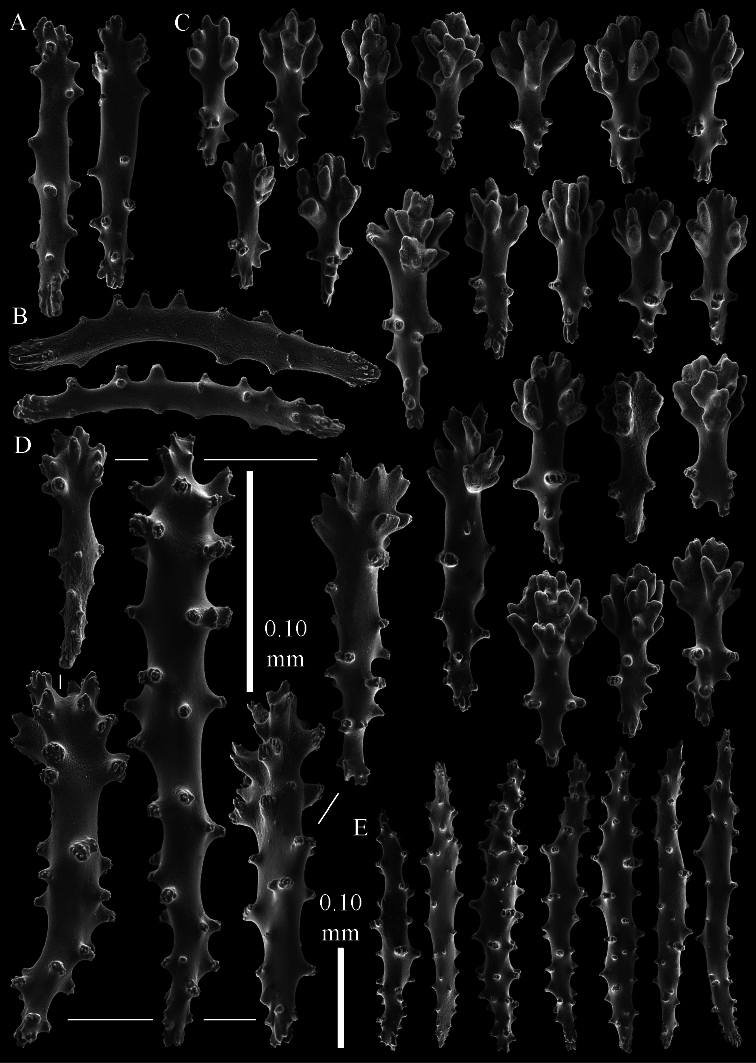
*Sinularia australiensis* sp. n., holotype NTM C14519. **A** point clubs **B** collaret spindles **C ***leptoclados*-type clubs of surface layer of lobule **D** wart clubs of surface layer of lobule **E** spindles of surface layer of lobule. Scale of 0.10 mm at **E** only applies to **E**.

The polyps have a collaret and eight points. Points with poorly developed clubs, up to 0.15 mm long (Fig. 2A). Collaret has bent spindles, up to 0.20 mm long (Fig. 2B). Tentacle sclerites were not present.

The surface layer of the lobules has *leptoclados*-type clubs, the smallest are 0.07 mm long, most are around 0.10 mm, but some even reach a length of 0.15 mm (Fig. 2C); in addition, longer wart clubs are present, up to 0.25 mm long (Fig. 2D). Furthermore, the surface layer of the lobules has spindles, up to 0.40 mm long, with simple tubercles (Fig. 2E).

**Figure 3. F3:**
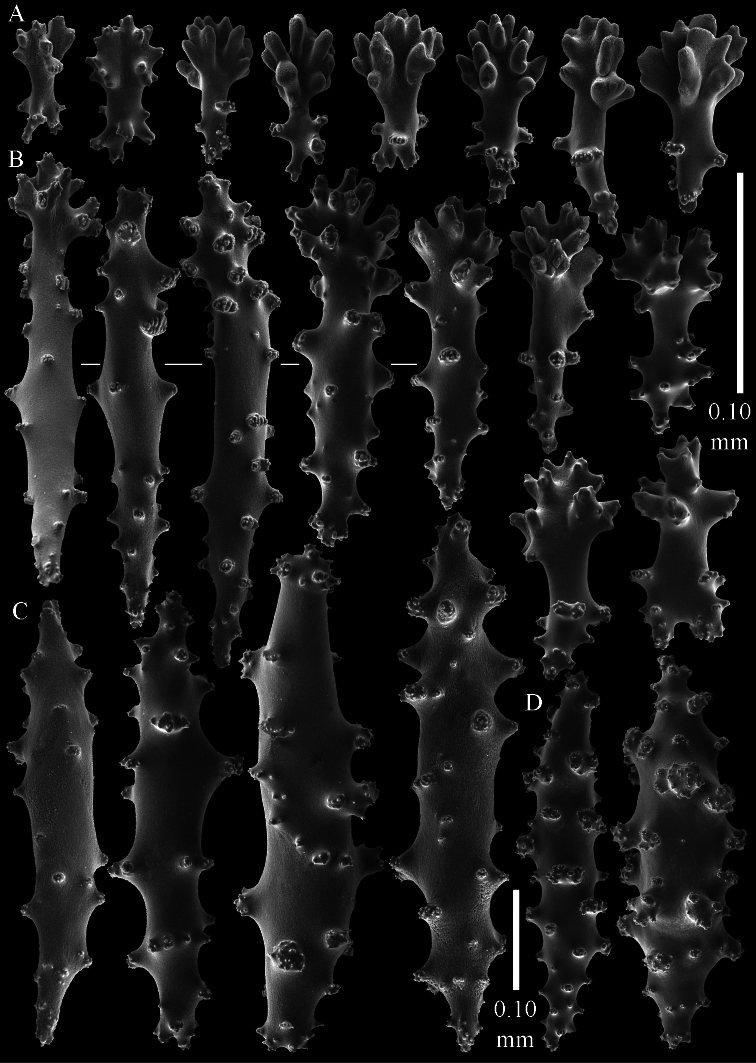
*Sinularia australiensis* sp. n., holotype NTM C14519. Sclerites of the surface layer of the base of the colony **A** *leptoclados*-type clubs **B** wart clubs **C–D** spindles. Scale of 0.10 mm at **D** only applies to **D**.

The sclerites of the surface layer of the base of the colony resemble those of the surface layer of the lobules but the clubs have wider handles and the spindles are wider (Fig. 3).

**Figure 4.**
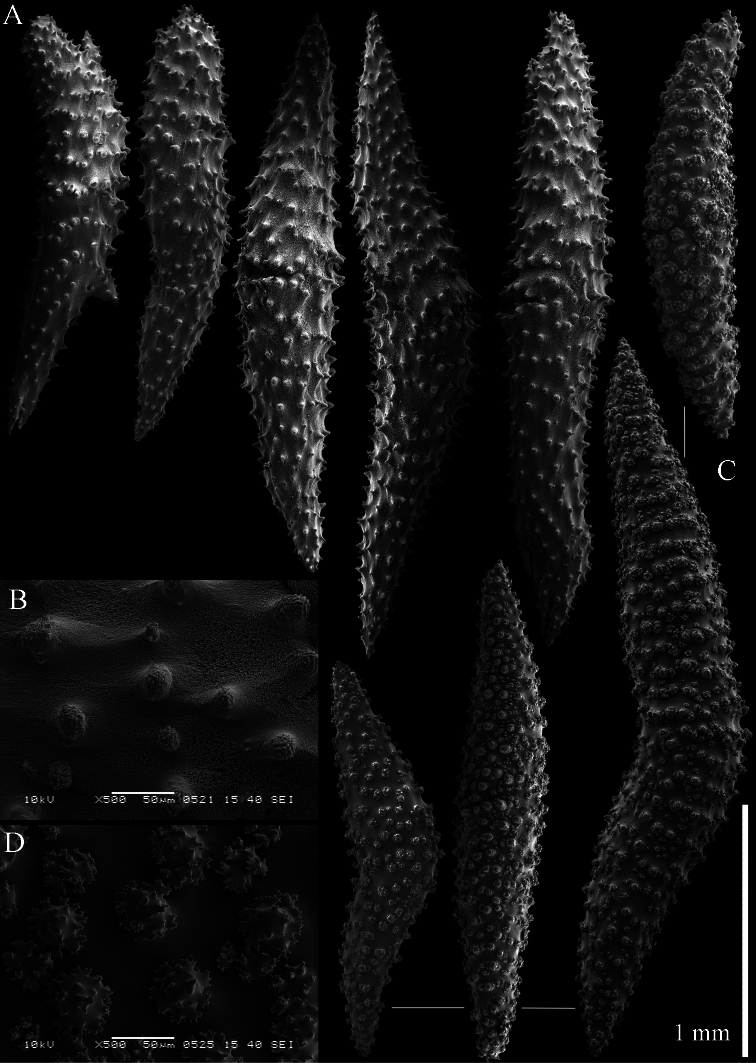
*Sinularia australiensis* sp. n., holotype NTM C14519. sclerites of the interior **A** spindles from the lobules **B** tuberculation of one of the lobule spindles **C** spindles from the base **D** tuberculation of one of the base spindles. Scale of 1 mm at **C** also applies to **A**.

The interior of the colony has mostly unbranched spindles; a few have one or two side branches. In the lobules the spindles are up to 2.5 mm long (Fig. 4A), almost all having simple tubercles (Fig. 4B). In the base of the colony they are up to 3 mm long (Fig. 4C), with more complex tubercles (Fig. 4D).

#### Colour.

The preserved specimen is brown.

#### Etymology.

Named after Australia, where the type was collected.

#### Intraspecific variation.

NTM C14492 (Fig. 1B) and NTM C14521 (Fig. 1D) have stouter lobules, up to 1 cm wide.

#### Remarks.

The species resembles *Sinularia leptoclados* regarding clubs and colony shape. It differs in having small surface lobule spindles with uniformly placed tubercles and many internal lobule spindles with simple tubercles. Other species resembling *Sinularia australiensis* are *Sinularia acuta* Manuputty & Ofwegen, 2007, S. *corpulentissima* Manuputty & Ofwegen, 2007 and *Sinularia longula* Manuputty & Ofwegen, 2007, all three described from Ambon. *Sinularia acuta* and *Sinularia longula* have more slender spindles and wart clubs in the surface layer of the lobules (Manuputty and Ofwegen 2007: Figs 3, 19). *Sinularia corpulentissima*, like *Sinularia leptoclados*, differs in having many internal spindles with complex tubercles (Manuputty and Ofwegen: Fig. 7c). Moreover, in the current molecular study *Sinularia corpulentissima* is assigned to a distinct subclade together with *Sinularia maxima*, while *Sinularia acuta* and *Sinularia longula* fall into a separate well-supported subclade (Figs 16, 17). *Sinularia australiensis* sp. n. does not belong to either of those subclades, but is close genetically to *Sinularia leptoclados* and *Sinularia abrupta*. The latter species has clubs resembling those of *Sinularia leptoclados* and *Sinularia australiensis*, but a totally different colony shape, with ridges instead of lobes with lobules.

Lüttschwager (1915) and Macfadyen (1936) had *Sinularia* material from Australia that could belong to *Sinularia australiensis*, but re-examination of sclerites of these specimens is necessary to confirm this possibility

### Sinularia eilatensis sp. n.

urn:lsid:zoobank.org:act:2DE6BD04-F415-48CB-AFB5-ABF3EF9BA63D

http://species-id.net/wiki/Sinularia_eilatensis

**Figure 5.**
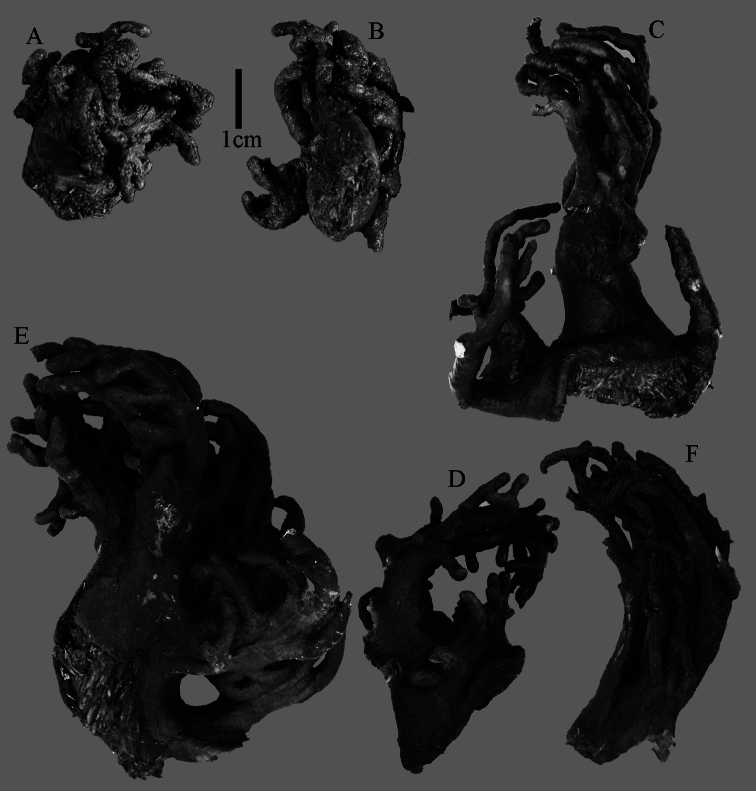
*Sinularia eilatensis* sp. n., colonies. **A** ZMTAU Co 35260, holotype **B** ZMTAU Co 35261, paratype **C** ZMTAU Co 35305, paratype **D** ZMTAU Co 35303 **E** ZMTAU Co 35304 **F** *Sinularia verseveldti*, ZMTAU Co 35309.

Figs 5A–E 6 [Fig F7] [Fig F8] –9

#### Type material examined.

holotype ZMTAU Co 35260, Israel, Red Sea, northern Gulf of Aqaba, Eilat, IUI (the Interuniversity Institute for Marine Sciences in Eilat) reef, depth 6 m, coll. Y. Benayahu, 10 January 2011; paratypes: ZMTAU Co 35261, same data as holotype; ZMTAU Co 35305, same data as holotype, 30 May 2011.

**Other material examined:** ZMTAU Co 35303-04, Israel, Red Sea, northern Gulf of Aqaba, Eilat, IUI reef, depth 5 m, coll. Y. Benayahu, 30 May 2011.

#### Description.

The holotype is 3.4 cm high and wide (Fig. 5A). The primary lobes branch off once or twice, lobules finger-shaped, up to 2 mm wide and 1 cm long.

**Figure 6.**
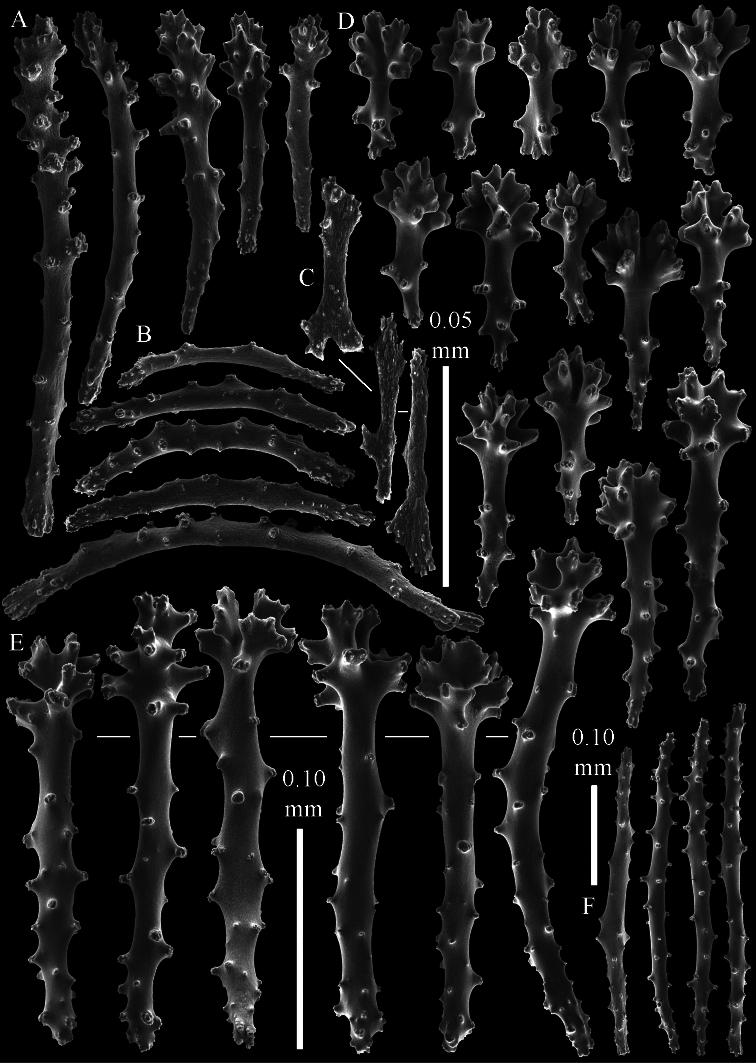
*Sinularia eilatensis* sp. n., holotype ZMTAU Co 35260. **A** point clubs **B** collaret spindles **C** tentacle rods **D** *leptoclados*-type clubs of surface layer of lobule **E** wart clubs of surface layer of lobule **F** spindles of surface layer of lobule. Scale of 0.10 mm at **F** only applies to **F**.

The polyps have a collaret and eight points. Points with poorly developed clubs, up to 0.25 mm long (Fig. 6A), collaret with bent spindles, up to 0.25 mm long (Fig. 6B) Tentacles with rods, about 0.05 mm long (Fig. 6C).

The surface layer of the lobules has *leptoclados*-type clubs, the smallest are 0.07 mm long, most are around 0.10 mm, but some reach a length of 0.15 mm (Fig. 6D); in addition longer wart clubs are present, up to 0.25 mm long (Fig. 6E). Furthermore, the surface layer of the lobules has spindles, up to 0.35 mm long, with simple tubercles (Fig. 6F).

**Figure 7. F7:**
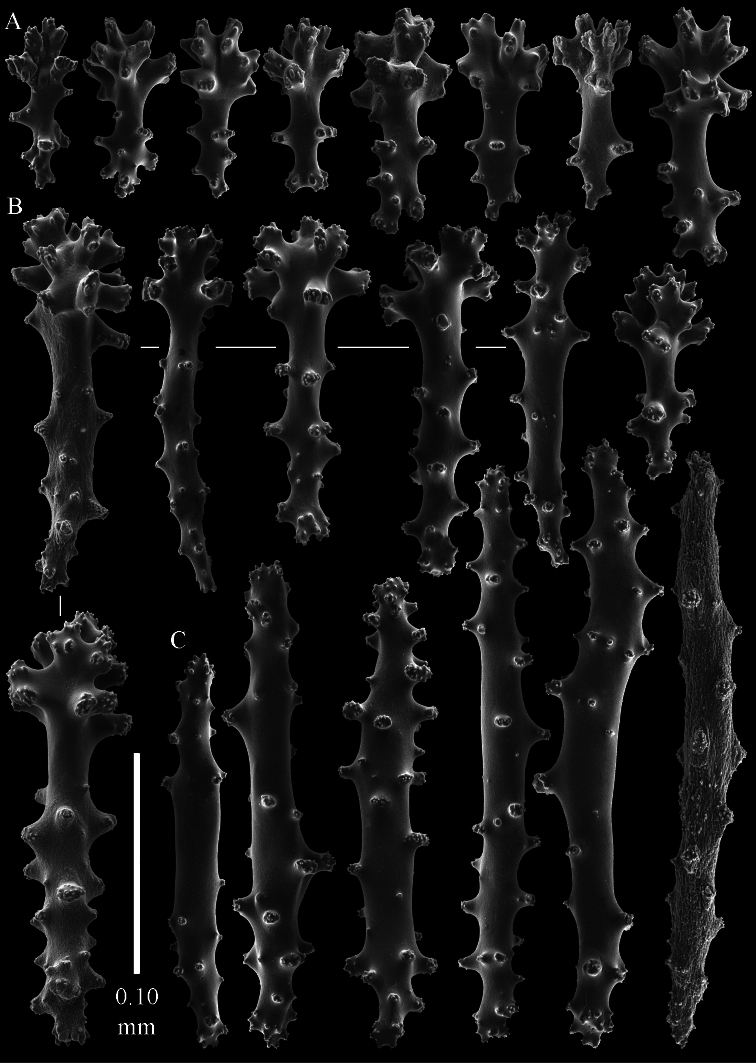
*Sinularia eilatensis* sp. n., holotype ZMTAU Co 35260. Sclerites of the surface layer of the base of the colony. **A** *leptoclados*-type clubs **B** wart clubs **C** spindles.

The sclerites of the surface layer of the base of the colony resemble those of the surface layer of the lobules but they are wider (Fig. 7).

**Figure 8. F8:**
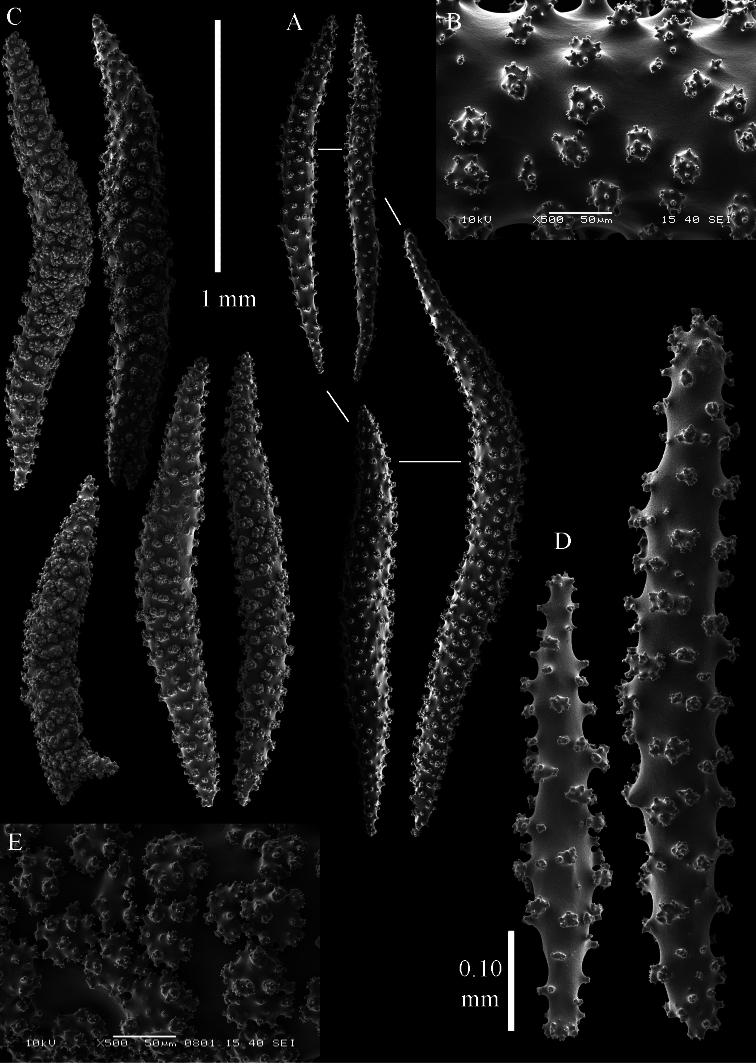
*Sinularia eilatensis* sp. n., holotype ZMTAU Co 35260. Sclerites of the interior **A** spindles from the lobules **B** tuberculation of one of the lobule spindles **C–D** spindles from the base **E** tuberculation of one of the base spindles. Scale at **D** only applies to **D**.

The interior of the colony has mostly unbranched spindles, a few have one or two side branches. In the lobules they are up to 2.5 mm long (Fig. 8A), with simple or complex tubercles (Fig. 8B). In the base of the colony the spindles are up to 2 mm long (Fig. 8C–D), with more complex tubercles (Fig. 8E).

**Figure 9.**
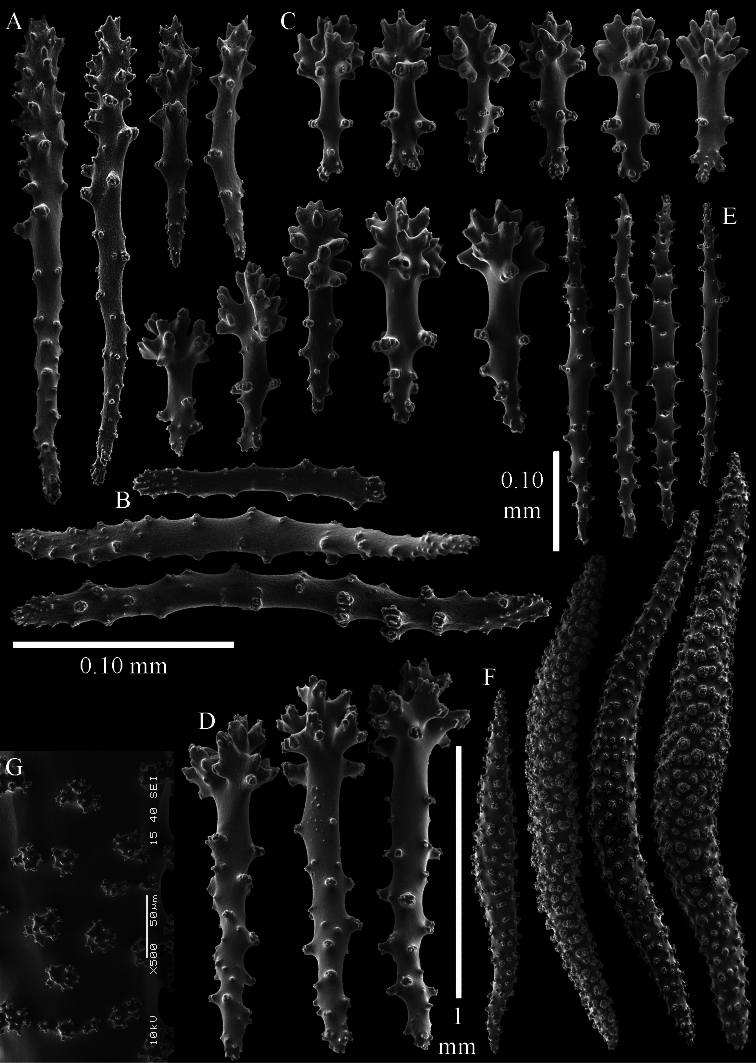
*Sinularia eilatensis* sp. n., ZMTAU Co 35304. **A** point clubs **B** collaret spindles **C** *leptoclados*-type clubs of surface layer of lobule **D** wart clubs of surface layer of lobule **E** spindles of surface layer of lobule **F** interior spindles of lobule **G** tuberculation of one of the lobule spindles. Scale of 0.10 mm at **E** only applies to **E**, 1 mm scale at **F** only to **F**.

#### Colour.

The preserved holotype is dark brown.

#### Etymology.

Named after Eilat, the type locality.

#### Intraspecific variation.

ZMTAU Co 35305 (Fig. 5C) has distinctly longer lobules, up to 2 cm long.

#### Remarks.

The speciesis unique among *Sinularia* species with *leptoclados*-type clubs by its very long point and collaret sclerites.

We excluded ZMTAU Co 35303-04 (Fig. 5D–E) from the type series. Morphologically we could not find a difference between these two specimens and the types, but their mitochondrial gene haplotypes differ by 0.5%. For comparison, we also present sclerites of ZMTAU Co 35304 (Fig. 9).

### Sinularia leptoclados

(Ehrenberg, 1834)

http://species-id.net/wiki/Sinularia_leptoclados

**Figure 10.**
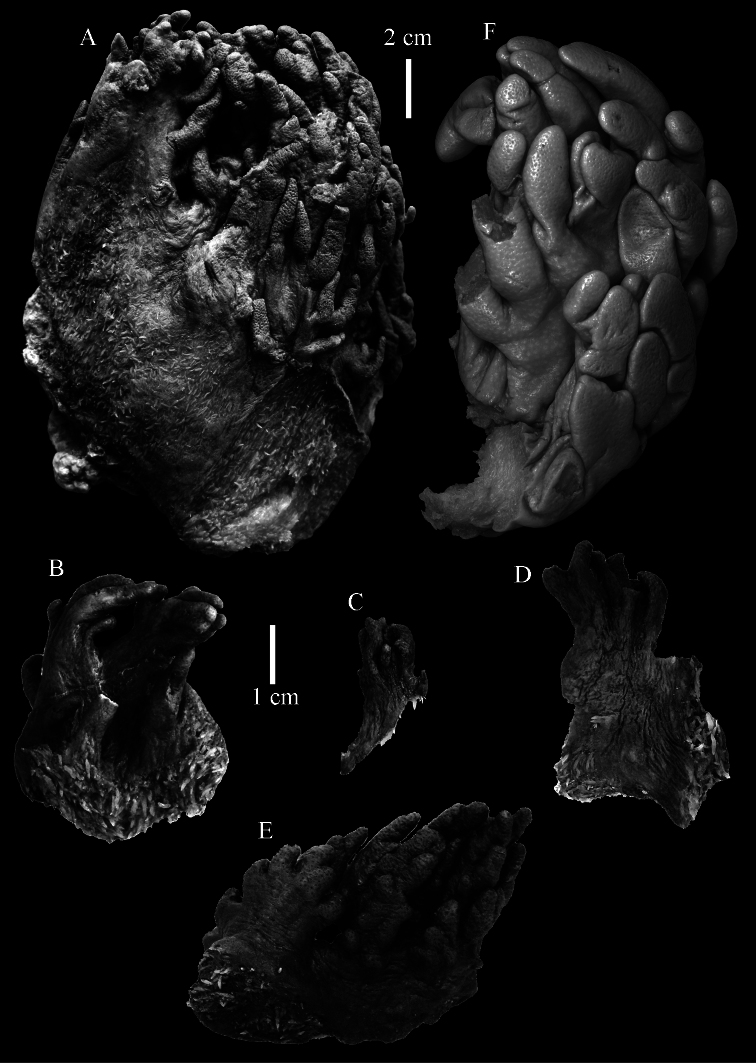
*Sinularia leptoclados* colonies. **A** ZMB 304 holotype **B** ZMTAU Co 34093 **C** ZMTAU Co 34094 **D** ZMTAU Co 34095 **E** ZMTAU Co 35308 **F** *Sinularia maxima*, ZRC1999.1066. Scale of 2 cm only applies to **A** and **F**.

Figs 10A–E 11 [Fig F12] [Fig F13] –14

Lobularia leptoclados Ehrenberg, 1834: 58 (Red Sea).
Alcyonium leptoclados ; Klunzinger 1877: 26, pl. 1 fig. 7a-d (Red Sea).
Sinularia leptoclados ; Tixier-Durivault 1951: 124, figs 173-175 (Red Sea); 1966: 218, 222, figs 212–214 (Madagascar); Verseveldt 1965: 29 (Red Sea); 1971: 4 (Madagascar); Ofwegen and Benayahu 1992: 140 (Tanzania); Benayahu and Schleyer 1996: 6 (Mozambique); Benayahu et al. 2002: 278 (Southern Red Sea).
NOT Alcyonium leptoclados ; Burchardt 1903: 661, pl. 54 fig. 6, pl. 56 fig. 4 (Torres Strait, Ambon).
NOT Sinularia leptoclados ; Thomson and Dean 1931: 45, pl. 11 fig. 5, pl. 21 figs 6, 9 (Indonesia); Roxas 1933: 350, pl. 2 fig. 8 (Philippines); Verseveldt 1974: 96 (New Caledonia); 1977: 3 (Gambier Island, Fanning Atoll, Enewetak); 1978: 50 (Guam); Ofwegen and Vennam 1994: 138 (Ambon, Indonesia); Benayahu 1993: 6 (South Africa); 1995: 107 (Ryukyu Archipelago, Japan); Ofwegen 1996: 208 (Bismarck Sea); Benayahu 1997: 210 (Guam); Benayahu 2002: 14 (Ryukyu Archipelago, Japan); Benayahu et al. 2004: 551 (Taiwan); Manuputty and Ofwegen 2007: 192, figs 2b, 5 (Ambon, Indonesia; = *Sinularia verseveldti*); Ofwegen 2008a: 131 (Gulf of Carpentaria, Australia; = *Sinularia australiensis* sp. n.).
NOT Sinularia aff. *leptoclados* Ofwegen, 2008b: 671 (Palau; = *Sinularia verseveldti*).
NOT Sinularia leptoclados var. *gonatodes* Kolonko, 1926: 309, pl. 2 fig. 1 (Philippines); Roxas 1933: 351 (same data as Kolonko) (= *Sinularia maxima* Verseveldt, 1971)
? Sclerophytum herdmanni Pratt, 1905: 235, pl. 2 figs 8–9 (Sri Lanka; needs re-examination).

#### Material examined.

ZMB 304, holotype of *Lobularia leptoclados* Ehrenberg; 1834, Rotes Meer, leg. Hemprich. Additional material: **Red Sea;** ZMTAU Co 25763, Egypt, Sinai, Tiran Strait, Thomas W., depth 3 m, coll. Y. Benayahu, 25 June 1985; ZMTAU Co 25940, Egypt, Gulf of Suez, Jubal Island, Bluf Point, depth 16 m, coll. Y. Benayahu, 24 March 1988; ZMTAU Co 34093-95, Israel, Gulf of Aqaba, Eilat, Nature Reserve, 29°30.6'N, 34°55.35'E, depth 2.4–5.5 m, coll. Y. Benayahu, 24 July 2007; ZMTAU Co 35308, Israel, Gulf of Aqaba, Eilat, Nature Reserve, depth 3 m, coll. Y. Benayahu, 31 May 2011; **Kenya;** ZMTAU Co 30354, off Mombasa, Shelly Reef, 04°07'S, 39°40'E, depth 12–13 m, coll. Y. Benayahu & S. Perkol, 20 January 2000; ZMTAU Co 32549, Shimoni, Wasini Is., opposite the building, depth 5 m, coll. Y. Benayahu, 2 February 2003; **Tanzania;** RMNH Coel. 18953, off Dar es Salaam, Pangavinne Island, seaward slope (P02), 6°50'S, 39°17'E, depth 6 m, coll. J.N. Nyanda; RMNH Coel. 18954, off Dar es Salaam, Pangavinne Island, seaward slope (P18), 6°50'S, 39°17'E, depth 8 m, coll. J.N. Nyanda; RMNH Coel. 18955, off Dar es Salaam, Mbudya Island, seaward slope (P35), 6°50'S, 39°17'E, depth 5 m, coll. J.N. Nyanda; ZMTAU Co 26314, Pangavinne Is., depth 6 m, coll. J.N. Nyanda, 1991; ZMTAU Co 26316, Mbudya Is., depth 5 m, coll. J.N. Nyanda, 1991; **Mozambique;** ZMTAU Co 28796, Bazaruto Is., Manta Reef, depth 15 m, coll. M. Schleyer, 7 October 1994; **Madagascar;** RMNH Coel. 6653, Ankify, on mainland of Madagascar, opposite Nosy Komba, depth 1 m, 22 July 1967, coll. A.G. Humes (1183); RMNH Coel. 6654, Ankify, on mainland of Madagascar, opposite Nosy Komba, depth 1 m, 11 August 1967, coll. A.G. Humes (1250); RMNH Coel. 6655, Ankify, on mainland of Madagascar, opposite Nosy Komba, depth 1 m, 23 August 1967, coll. A.G. Humes (1320); RMNH Coel. 6659, Nosy Iranja, SW Nosy Bé, depth 15 m, 9 August 1967, coll. A.G. Humes (1239); RMNH Coel. 6660, W of Andilana, 13°18'S, 48°07'E, 20 m deep, 24 August 1967, coll. A.G. Humes (1331); RMNH Coel. 6656, Ankify, on mainland of Madagascar, opposite Nosy Komba, depth 1 m, 23 August 1967, coll. A.G. Humes (1321); RMNH Coel. 6657, Ankify, on mainland of Madagascar, opposite Nosy Komba, depth 1 m, 23 August 1967, coll. A.G. Humes (1322); RMNH Coel. 6658, Ankify, on mainland of Madagascar, opposite Nosy Komba, depth 1 m, 23 August 1967, coll. A.G. Humes (1323); RMNH Coel. 6661, Pass at Pte Lokobe, Nosy Bé, Madagascar, depth 15 m, 19 June 1967, coll. A.G. Humes (A28).

#### Description.

The holotype is 18 cm high and 13 cm wide (Fig. 10A). The primary lobes branch off once or twice, lobules finger-shaped, up to 1 cm wide and 3 cm long.

**Figure 11.**
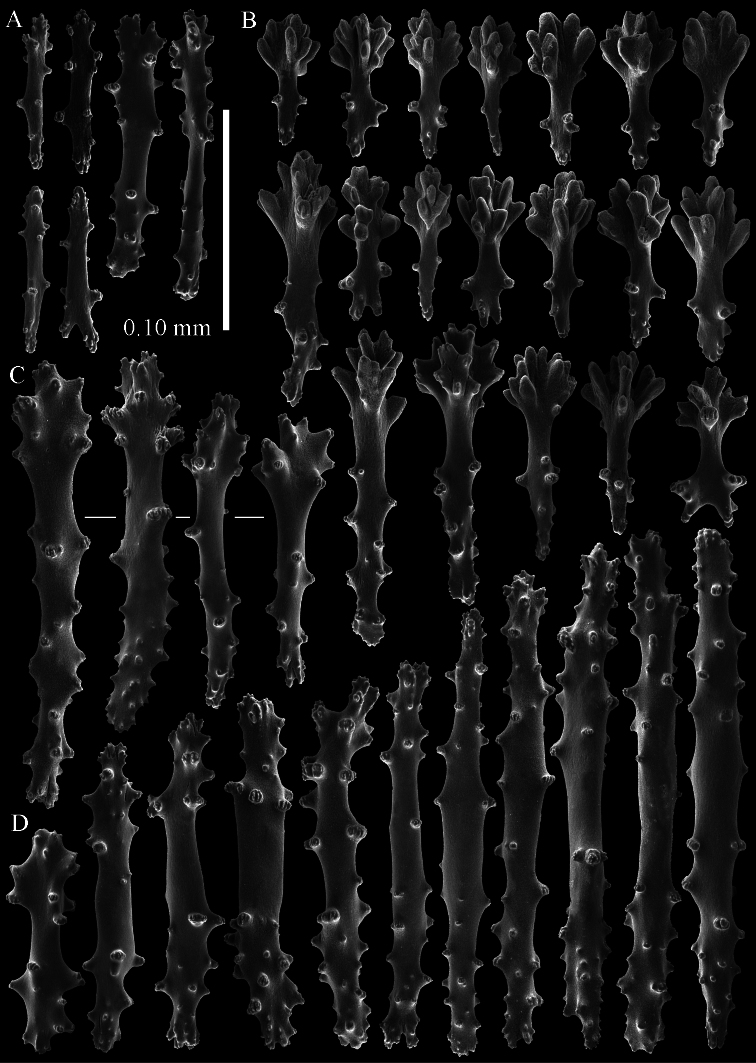
*Sinularia leptoclados* holotype ZMB 304. **A** point clubs **B** *leptoclados*-type clubs of surface layer of lobule **C** wart clubs of surface layer of lobule **D** spindles of surface layer of lobule.

The polyps have a collaret and eight points. Points with poorly developed clubs, up to 0.13 mm long (Fig. 11A), collaret with bent spindles. Tentacle sclerites not observed.

The surface layer of the lobules has *leptoclados*-type clubs, the smallest are 0.05 mm long, most are around 0.10 mm, but some reach a length of 0.15 mm (Fig. 11B); in addition longer wart clubs are present, up to 0.20 mm long (Fig. 11C). Furthermore, the surface layer of the lobules has spindles, up to 0.45 mm long, with simple tubercles (Fig. 11D, 12A); the smaller ones with a distinct median waist.

The interior of the colony has unbranched spindles. In the lobules they are up to 2.5 mm long (Fig. 12B), with simple or complex tubercles (Fig. 12C). In the base of the colony the spindles are also up to 2 mm long (Fig. 12D), with more complex tubercles (Fig. 12E).

**Figure 13. F13:**
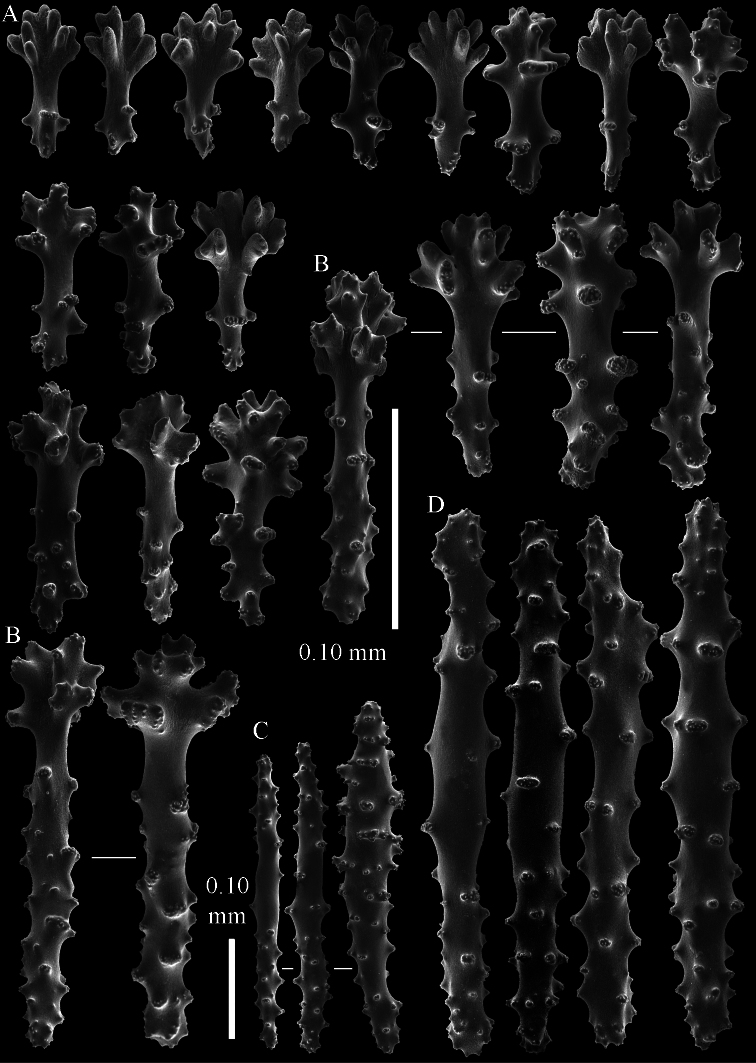
*Sinularia leptoclados* holotype ZMB 304. Sclerites of the surface layer of the base of the colony. **A** *leptoclados*-type clubs **B** wart clubs **C–D** spindles. Scale of 0.10 mm at **C** only applies to **C**.

The sclerites of the surface layer of the base of the colony resemble those of the surface layer of the lobules but they are wider (Fig. 13).

**Figure 12. F12:** *Sinularia leptoclados* holotype ZMB 304. **A** spindles of the surface layer of lobule **B–D** sclerites of the interior **B** spindles from the lobules **C** tuberculation of one of the lobule spindles **D** spindles from the base **E** tuberculation of two of the base spindles. Scale of 0.10 mm at **A** only applies to **A**.

**Figure 14.**
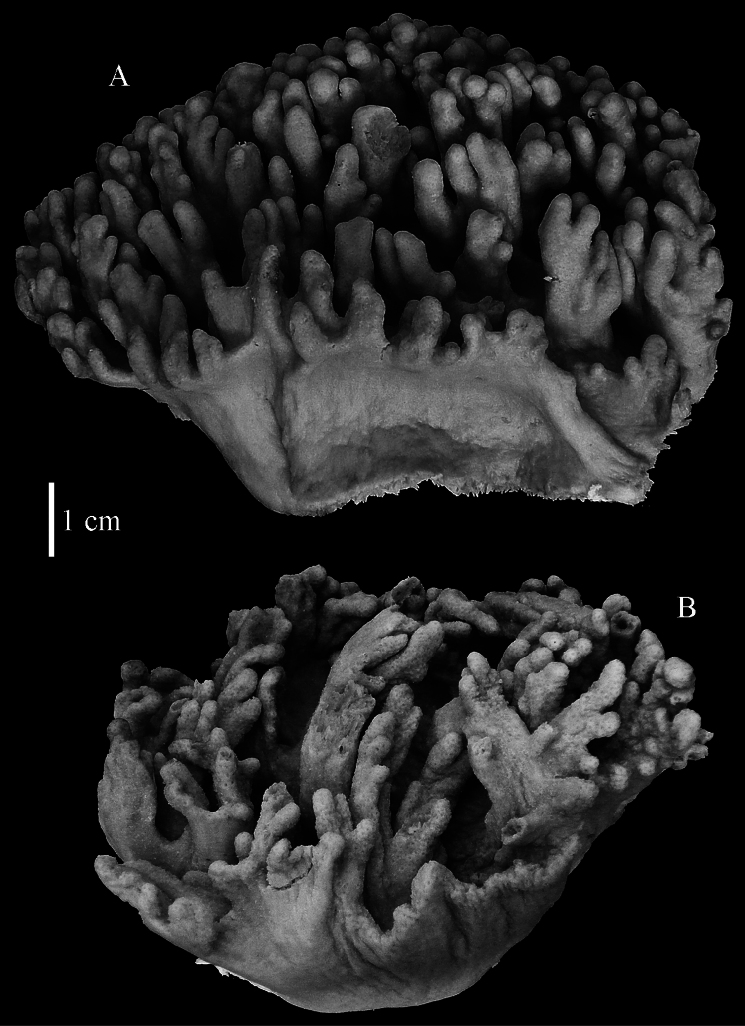
*Sinularia leptoclados* colonies. **A** ZMTAU Co 25763 **B** ZMTAU Co 25940.

#### Colour.

The holotype is brown.

#### Intraspecific variation.

Most of the colonies of *Sinularia leptoclados* are stalked and rarely feature an encrusting colony shape (Fig. 14).

#### Remarks.

**Figure 15.**
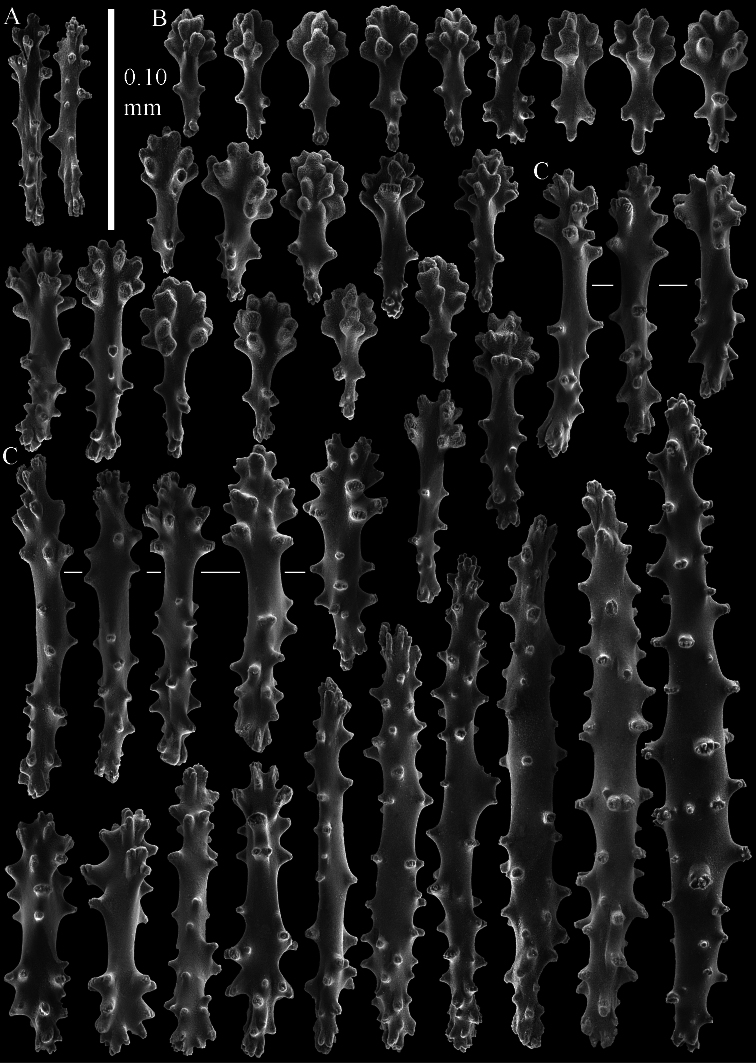
*Sinularia leptoclados* var. *gonatodes* ZMB 6495 **A** point clubs **B** *leptoclados*-type clubs of surface layer of lobule **C** wart clubs of surface layer of lobule **D** spindles of surface layer of lobule.

Verseveldt (1980) re-examined ZMB 6495, the type specimen of Kolonko’s *Sinularia leptoclados* var. *gonatodes* from the Bata islands (East coast of Palawan, Philippines), and considered it nothing else than *Sinularia leptoclados* (Ehrenberg, 1834). Alderslade and Shirwaiker (1991) also re-examined ZMB 6495, assuming it was *Sinularia leptoclados*, to differentiate their *Sinularia kavarattiensis*. They noticed many small rods in the surface layer of the lobes. Unfortunately, neither Verseveldt nor Alderslade and Shirwaiker presented any figures of sclerites of *Sinularia leptoclados* var. *gonatodes*. Here we present such sclerites of the lobe surface (Fig. 15), which are more like those of *Sinularia maxima* Verseveldt, 1971, and therefore we consider *Sinularia leptoclados* var. *gonatodes* to be *Sinularia maxima*. The main difference between *Sinularia leptoclados* and *Sinularia maxima* is not in the sclerites but concerns the much wider lobes of the latter (Fig. 10E). As a consequence, Alderslade and Shirwaiker (1991) compared their S. *kavarattiensis* with *Sinularia maxima* instead of with *Sinularia leptoclados*. Their new species differs from both in having *leptoclados*-type clubs with an angle between the head and handle of about 90 degrees, thus considered to be valid.

One other species that can be confused with *Sinularia leptoclados* is *Sinularia verseveldti* Ofwegen, 1996. Its colony shape was described as being cup-shaped, but examination of many specimens from Indonesia showed that colony shape to be exceptional. Mostly the colonies resemble *Sinularia leptoclados* very closely. Manuputty and Ofwegen (2007, fig. 2b, fig. 5) showed such a colony and its sclerites. The species differs in club shape, with the angle between the head and handle larger than 90 degrees in *Sinularia leptoclados* and about 90 degrees in *Sinularia verseveldti*. *Sinularia* aff. *leptoclados* in Ofwegen (2009: 671) we now consider also to be *Sinularia verseveldti*.

## Molecular Results

**Figure 16.**
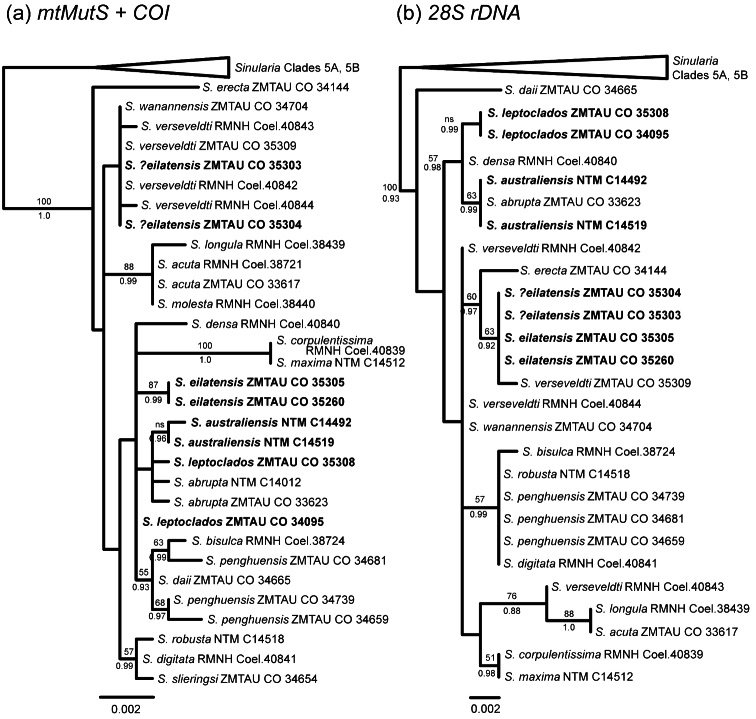
Maximum likelihood trees of *Sinularia* clade 5C (McFadden et al. 2009) based on (**a**) combined analysis of two mitochondrial genes (*mtMutS*, *COI*), and (**b**) nuclear 28S rDNA. Specimens described in this publication in bold. Numbers above branches are bootstrap values from maximum likelihood analysis (only values >50% shown; ns = value <50%); numbers below branches are Bayesian posterior probabilities (only values > 0.85 shown).

Sequences for *mtMutS* and *COI* (including *igr1*) were available or newly obtained for 31 specimens representing 19 morphospecies of *Sinularia* belonging to clade 5C; 28S rDNA sequences were obtained for all but four specimens (Table 1). *mtMutS* (735 nt) and *COI* (888 nt) sequences were concatenated for a total mitochondrial gene alignment of 1623 nt. 28S sequences ranged from 797–799 nt in length for a total alignment length of 801 nt. Maximum likelihood and Bayesian analyses resulted in identical tree topologies for all three data sets (mt genes only, 28S only, all three genes combined). Support values were generally somewhat stronger for Bayesian analyses, however, and several nodes that were not supported by maximum likelihood (bootstrap values <50%) nonetheless had Bayesian posterior probabilities >0.9 (Figs 16, 17). All alignments and trees have been submitted to TreeBASE (www.treebase.org ).

Within *Sinularia* species with *leptoclados*-type clubs (clade 5C), genetic distances (uncorrected p) among recognized morphospecies range from only 0–1.7% for *mtMutS*, 0–0.8% for *COI* and 0–1.4% for 28S rDNA. Despite these relatively low levels of genetic differentiation among taxa, several moderately- to well-supported clades appear in both the mitochondrial and 28S gene trees (Fig. 16). *Sinularia maxima* and *Sinularia corpulentissima* share identical mt and 28S haplotypes with one another, but are well differentiated from all other species in clade 5C. *Sinularia acuta*, *Sinularia longula* and *Sinularia molesta* are also very similar to one another genetically (*Sinularia molesta* and *Sinularia acuta* share identical mt and 28S haplotypes), and form a well-supported clade in both trees. Finally, *Sinularia erecta* is genetically distinct, separated from all other species by genetic distances of >0.8% at *mtMutS* (28S was not available for *Sinularia erecta*).

Two additional clades are moderately supported by the combined analysis of the mt and 28S genes (Fig. 17); the species in these clades also group together in the separate analyses, but with low bootstrap support (<50%) (Fig. 16). *Sinularia penghuensis*, *Sinularia bisulca*, *Sinularia robusta*, *Sinularia digitata* and *Sinularia slieringsi* comprise one of these moderately-supported clades (Fig. 17); these species share identical or nearly identical 28S sequences (28S was not available for *Sinularia slieringsi*) (Fig. 16b). Within the mt gene tree (Fig. 16a) they constitute two distinct clades, one comprised by *Sinularia robusta*, *Sinularia digitata* and *Sinularia slieringsi* and the other by *Sinularia penghuensis*, *Sinularia bisulca* and *Sinularia daii*. The latter is, however, distinct from all other species at 28S, and falls outside of this clade in the combined analysis (Fig. 17). *Sinularia leptoclados*, *Sinularia abrupta*, *Sinularia australiensis* sp. n. and *Sinularia densa* also form a moderately-supported clade in the 28S tree (Fig. 16b) and in the combined tree (supported by Bayesian but not maximum likelihood analyses; Fig. 17), but their relationship is unresolved in the mt tree (Fig. 16a). *Sinularia australiensis* sp. n. and *Sinularia abrupta* share identical 28S haplotypes, but differ from *Sinularia leptoclados* by 0.3%. *Sinularia australiensis* sp. n. differs from both *Sinularia leptoclados* and *Sinularia abrupta* by 0.1% and 0.1-0.2% at *mtMutS* and *COI* respectively.

The relationships among the remaining species in the clade — *Sinularia verseveldti*, *Sinularia wanannensis* and *Sinularia eilatensis* sp. n. — were poorly resolved and exhibited some incongruence between the mitochondrial and 28S gene trees. *Sinularia wanannensis*, all four specimens of *Sinularia verseveldti*, and two specimens (ZMTAU Co 35303, ZMTAU Co 35304) that were tentatively assigned to *Sinularia eilatensis* sp. n. share identical or nearly identical *mtMutS* and *COI* haplotypes, and cluster together within the mt tree (but with bootstrap values <50%). Two specimens of *Sinularia eilatensis* sp. n. (ZMTAU Co 35305, ZMTAU Co 35260) fall outside of that group, and differ from it by >0.5% at *mtMutS* (Fig. 16a). At 28S, however, ZMTAU Co 35303 and ZMTAU Co 35304 are genetically identical to both individuals of *Sinularia eilatensis* sp. n., and those four specimens form a moderately-supported clade together with *Sinularia verseveldti* ZMTAU Co 35309 (Fig. 16b). Two additional specimens of *Sinularia verseveldti* share identical 28S haplotypes with *Sinularia wanannensis*. The combined tree reflects the topology of the mt gene tree, and shows the separation of *Sinularia eilatensis* sp. n. (ZMTAU Co 35305, ZMTAU Co 35260) from ZMTAU Co 35303, ZMTAU Co 35304 and all other species (Fig. 17).

Our findings indicate that specimens of the same species generally shared identical or nearly identical sequences at all three loci. The only exceptions were the two distinct mitochondrial haplotypes of *Sinularia eilatensis* sp. n. discussed above, and the four specimens of *Sinularia verseveldti*. All *Sinularia verseveldti* shared identical or nearly identical *mtMutS* and *COI* sequences, but differed at 28S. Most of these differences, however, reflected polymorphic nucleotide positions at which one or more specimens exhibited heterozygosity. For example, at position 533 of the 28S alignment, ZMTAU Co 35309 and Coel. 40842 had C, Coel. 40843 had T, and Coel. 40844 had both C and T. A total of 8 such heterozygous nucleotide sites among the four *Sinularia verseveldti* specimens contribute to their disjunct distribution within the 28S and combined trees.

**Figure:**
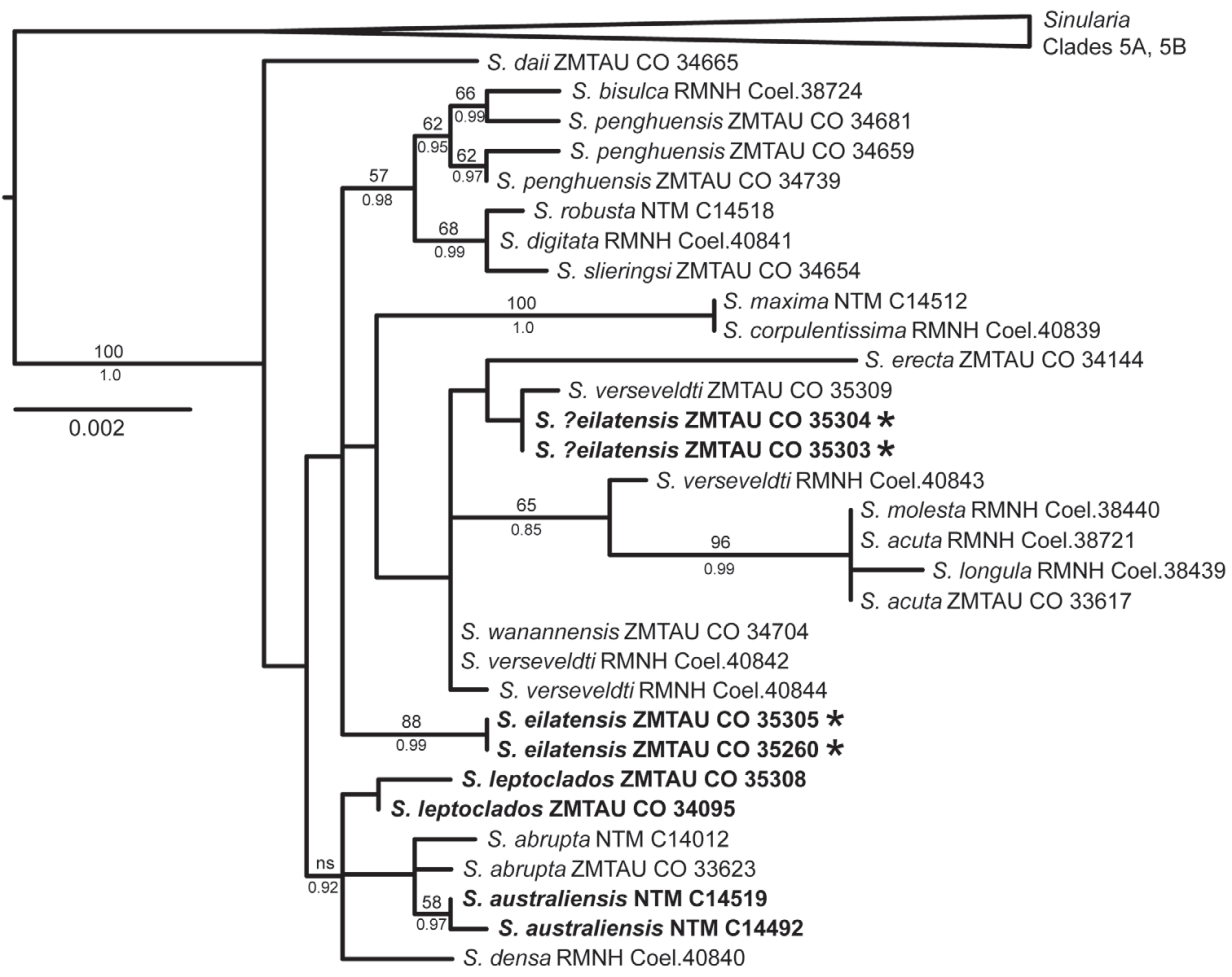
**Figure 17.** Maximum likelihood tree of *Sinularia* clade 5C (McFadden et al. 2009) based on a combined, partitioned analysis of two mitochondrial genes (*mtMutS, COI*) and nuclear 28S rDNA. Specimens described in this publication in bold. Specimens indicated with * have different mtDNA haplotypes but identical 28S rDNA sequences. Numbers above branches are bootstrap values from maximum likelihood analysis (only values >50% shown; ns = value <50%); numbers below branches are Bayesian posterior probabilities (only values > 0.85 shown).

## Discussion

The two new species described here are supported both by morphological characters and by the molecular analysis. Although *Sinularia australiensis* sp. n. is similar genetically to *Sinularia leptoclados* and both belong to the same sub-clade within *Sinularia* clade 5C, they differ at all three of the loci sequenced here. Furthermore, the 28S and combined analyses suggest that *Sinularia leptoclados* and *Sinularia australiensis* sp. n. are not sister taxa, but that *Sinularia australiensis* sp. n. is closer to *Sinularia abrupta*, a species with which it shares a 28S haplotype. The disjunct geographical distribution between *Sinularia leptoclados*, which occurs in the Red Sea and western Indian Ocean, and *Sinularia australiensis* sp. n. from Australia, further supports their distinction.

Although sympatric with *Sinularia leptoclados* in the Red Sea, *Sinularia eilatensis* sp. n. is clearly distinct from that species, both morphologically and genetically. Within clade 5C, *Sinularia eilatensis* sp. n. is most similar genetically to the geographically widespread *Sinularia verseveldti* and to *Sinularia wanannensis*, a species recently described from Taiwan (Ofwegen and Benayahu 2012). Morphologically, however, *Sinularia eilatensis* clearly differs from *Sinularia verseveldti* and *Sinularia wanannensis* by its long polyp sclerites, up to 0.25 mm long in *Sinularia eilatensis* vs up to 0.15 mm long in the other two species. Undoubtedly, the phylogenetic relationships among these three species need further investigation. In particular, the conflicting phylogenetic signals obtained from the mitochondrial and nuclear genes suggest the possibility of past hybridization events between *Sinularia verseveldti* and *Sinularia eilatensis* sp. n. Two specimens from the Red Sea (ZMTAU Co 35030 and ZMTAU Co 35304) appear morphologically to belong to *Sinularia eilatensis* sp. n. and have the same 28S sequence as that species but share a distinct mitochondrial haplotype with *Sinularia verseveldti*. This observed mito-nuclear discord could reflect a hybrid origin of these specimens, as has been suggested for some other octocorals (reviewed in McFadden et al. 2010). In addition, the polymorphism observed at the 28S locus in *Sinularia verseveldti* could be indicative of recent hybridization events involving this species, although it could also be the result of incomplete lineage sorting following recent speciation. The possible hybrid origin of ZMTAU Co 35303 and ZMTAU Co 35304 should be investigated further using single-copy nuclear gene markers.

Previous molecular systematic work on *Sinularia* and other octocoral genera has highlighted the inadequacies of mitochondrial gene markers for species discrimination and species-level phylogenetic analyses in the group (McFadden et al. 2009, 2011). Although both *mtMutS* and *COI* effectively distinguish genera and distinct clades within genera, neither gene is variable enough to distinguish all congeneric species pairs unequivocally. The region of the nuclear 28S rDNA gene we sequenced exhibits somewhat greater variability than *mtMutS* in some genera of the family Alcyoniidae (Benayahu et al. in press), but did not distinguish among all of the morphospecies of *Sinularia* examined in the current study. Despite the relatively small genetic distances separating morphospecies and the low resolution of the resulting phylogenies, we believe the analysis presented here adequately supports the distinctions of the new species that are the focus of this study. Development of additional, more variable molecular markers, will be necessary in order to fully resolve the relationships among morphospecies in *Sinularia* clade 5C and to address the possibility of hybridization among them.

## Supplementary Material

XML Treatment for Sinularia australiensis

XML Treatment for Sinularia eilatensis

XML Treatment for Sinularia leptoclados

## References

[B1] Alderslade P Shirwaiker P (1991) New species of soft corals (Coelenterata: Octocorallia) from the Laccadive Archipelago. Beagle 8 (1): 189-233.

[B2] Benayahu Y (1993) Corals of the South-west Indian Ocean. I. Alcyonacea from Sodwana Bay, South Africa. Investigational Report Oceanographic Research Institute 67: 1-16.

[B3] Benayahu Y (1995) Species composition of soft corals (Octocorallia, Alcyonacea) on the coral reefs of Sesoko Island, Ryukyu Archipelago, Japan. Galaxea 12: 103-124. doi: 10.3755/jcrs.2002.11

[B4] Benayahu Y (1997). A review of three alcyonacean families (Octocorallia) from Guam. Micronesia 30 (2): 207-244.

[B5] Benayahu Y (2002) Soft corals (Octocorallia: Alcyonacea) of the southern Ryukyu Archipelago: The families Tubiporidae, Clavulariidae, Alcyoniidae and Briareidae. Galaxea JCRS 4: 11-32.

[B6] Benayahu Y Schleyer MH (1996) Corals of the south-west Indian Ocean 3. Alcyonacea (Octocorallia) of Bazaruto Island, Mozambique, with a redescription of *Cladiella australis* (Macfadyen, 1936) and a description of *Cladiella kashmani* spec. nov. Oceanographic Research Institute Investigational Reports 69: 1-22.

[B7] Benayahu Y Jeng MS Perkol-Finkel S Dai CF (2004) Soft corals (Octocorallia: Alcyonacea) from southern Taiwan. II. Species diversity and distributional patterns. Zoological Studies 43 (3): 548-560.

[B8] Benayahu Y Ofwegen LP van Soong K Dai CF Jeng MS Shlagman A Hsieh HJ McFadden CS (in press) Diversity and distribution of Octocorals (Coelenterata: Anthozoa) on the coral reefs of Penghu, Taiwan. Zoological Studies.

[B9] Benayahu Y Tesfamariam Y Schleyer MH (2002) Soft corals (Octocorallia, Alcyonacae) of the southern Red Sea. Israel Journal of Zoology 48: 273-283. doi: 10.1560/HYC7-TUTH-EV77-BEUQ

[B10] Burchardt E (1903). Alcyonaceen von Thursday Island (Torres-Strasse) und von Amboina II. In: Semon R (Ed.). Zoologische Forschungsreisen in Australien und dem Malayischen Archipel 5 (6). Denkschriften der Medisch Naturwissenschaftlich Gesellschaft Jena 8: 654–682.

[B11] Ehrenberg CG (1834) Beiträge zur physiologischen Kenntniss der Corallenthiere im allgemeinen, und besonders des rothen Meeres, nebst einem Versuche zur physiologischen Systemat ik derselben. Abhandlungen der Königlichen Akademie der Wissenschaften zu Berlin Aus dem Jahre 1832 Erster Theil: 225–380.

[B12] Katoh K Kuma K Toh H Miyata T (2005) MAFFT version 5: improvement in accuracy of multiple sequence alignment. Nucleic Acids Research 33: 511-513. doi: 10.1093/nar/gki198 15661851 PMC548345

[B13] Klunzinger CB (1877) Die Korallthiere des Rothen Meeres. I. Die Alcyonarien und Malacodermen. Berlin.

[B14] Kolonko K (1926) Beitrage zu einer Revision der Alcyonarien. Die Gattung *Sinularia*. Mitteilungen aus dem Zoologischen Museum in Berlin 12 (2): 291-334.

[B15] Lüttschwager J (1915) Beiträge zu einer Revision der Familie Alcyoniidae. Archiv für Naturgeschichte 1914 A(10): 1–42.

[B16] MacFadyen LMI (1936) Alcyonaria (Stolonifera, Alcyonacea, Telestacea and Gorgonacea). Scientific Reports Great Barrier Reef Expedition 5 (2): 19-72.

[B17] Manuputty AEW Ofwegen LP van (2007) The genus *Sinularia* (Octocorallia: Alcyonacea) from Ambon and Seram (Moluccas, Indonesia). Zoologische Mededelingen Leiden. 81 (11): 187-216.

[B18] McFadden CS Ofwegen LP van (2012) A second, cryptic species of the soft coral genus *Incrustatus* (Anthozoa: Octocorallia: Clavulariidae) from Tierra del Fuego, Argentina revealed by DNA barcoding. Helgoland Marine Research, doi: 10.1007/s10152-012-0310-7

[B19] McFadden CS Ofwegen LP van Beckman EJ Benayahu Y Alderslade P (2009) Molecular systematics of the speciose Indo-Pacific soft coral genus, *Sinularia* (Anthozoa: Octocorallia). Invertebrate Biology 128: 303-323. doi: 10.1111/j.1744-7410.2009.00179.x

[B20] McFadden CS Sánchez JA France SC (2010) Molecular phylogenetic insights into the evolution of Octocorallia: a review. Integrative and Comparative Biology 50 (3): 389-410. doi: 10.1093/icb/icq056 21558211

[B21] Ofwegen LP van (1996) Octocorallia from the Bismarck Sea (part II). Zoologische Mededelingen Leiden 70 (13): 207-215.

[B22] Ofwegen LP van (2008a) The genus *Sinularia* (Octocorallia: Alcyonacea) from Bremer and West Woody Islands (Gulf of Carpentaria, Australia). Zoologische Mededelingen Leiden 82 (16): 131-165.

[B23] Ofwegen LP van (2008b) The genus *Sinularia* (Octocorallia: Alcyonacea) at Palau, Micronesia. Zoologische Mededelingen Leiden 82 (51): 631-735.

[B24] Ofwegen LP van Benayahu Y (1992) Notes on Alcyonacea (Octocorallia) from Tanzania. Zoologische Mededelingen Leiden 66 (6): 139-154.

[B25] Ofwegen LP van Benayahu Y (2012) Two new species and a new record of the genus *Sinularia* (Octocorallia, Alcyonacea) from Penghu archipelago, Taiwan. Zoological Studies51: 283–398.

[B26] Ofwegen LP Vennam J (1994). Results of the Rumphius Biohistorical Expedition to Ambon (1990). Part 3. The Alcyoniidae (Octocorallia: Alcyonacea). Zoologische Mededelingen Leiden 68 (14): 135-158.

[B27] Posada D Crandall KA (1998) Modeltest: testing the model of DNA substitution. Bioinformatics 14: 817-818. doi: 10.1093/bioinformatics/14.9.817 9918953

[B28] Pratt EM (1905) Report on some Alcyoniidae collected by Professor Herdman, at Ceylon, in 1902. Report to the Government of Ceylon on the Pearl Oyster Fisheries of the Gulf of Manaar 3 (Supplementary Report No. 19): 247–26.

[B29] Ronquist F Teslenko M van der Mark P Ayres D Darling A Höhna S Larget B Liu L Suchard MA Huelsenbeck JP (2012) MrBayes 3.2: Efficient Bayesian phylogenetic inference and model choice across a large model space. Systematic Biology 61: 539-542. doi: 10.1093/sysbio/sys029 22357727 PMC3329765

[B30] Roxas HA (1933) Philippine Alcyonaria, II. The Families Alcyoniidae and Nephthyidae. Philippine Journal of Science 50 (4): 345-470.

[B31] Tamura K Peterson D Peterson N Stecher G Nei M Kumar S (2011) MEGA5: Molecular evolutionary genetics analysis using maximum likelihood, evolutionary distance, and maximum parsimony methods. Molecular Biology and Evolution 28: 2731-2739. doi: 10.1093/molbev/msr121 21546353 PMC3203626

[B32] Tixier-Durivault A (1951) Révision de la famille des Alcyoniidae. Le genre *Sinularia* May, 1898. Mémoires de l’Institut Royal des Sciences Naturelles de Belgique (2)40: 1–146.

[B33] Tixier-Durivault A (1966) Octocoralliaires de Madagascar et des îles avoisinantes. Faune Madagascar 21: 1-456.

[B34] Thomson JA Dean LM (1931). The Alcyonacea of the Siboga Expedition with an addendum to the Gorgonacea. Siboga Expedition Monograph 13d: 1–227.

[B35] Verseveldt J (1965) Report on the Octocorallia (Stolonifera and Alcyonacea) of the Israel South Red Sea expedition 1962, with notes on other collections from the Red Sea. Sea Fisheries Research Station, Haifa, Bulletin 40 (Israel South Red Sea expedition 1962, reports): 28–48.

[B36] Verseveldt J (1971) Octocorallia from north-western Madagascar (Part 2). Zoologische Verhandelingen Leiden 117: 1-73.

[B37] Verseveldt J (1974) Octocorallia from New Caledonia. Zoologische Mededelingen Leiden 48: 95–122.

[B38] Verseveldt J (1977) Octocorallia from various localities in the Pacific Ocean. Zoologische Verhandelingen Leiden 150: 1-42.

[B39] Verseveldt J (1978) Alcyonaceans (Coelenterata: Octocorallia) from some Micronesian Islands. Zoologische Mededelingen Leiden 53: 49-55.

[B40] Verseveldt J (1980) A revision of the genus *Sinularia* May (Octocorallia, Alcyonacea). Zoologische Verhandelingen Leiden 179: 1-128.

[B41] Zwickl DJ (2006) Genetic algorithm approaches for the phylogenetic analysis of large biological sequence datasets under the maximum likelihood criterion. Ph.D. dissertation, University of Texas, Austin.

